# Alcoholism; Its Control and Cure in Sanatoriums

**Published:** 1902-07

**Authors:** George H. McMichael

**Affiliations:** Buffalo, N.Y.


### Alcoholism; Its Control and Cure in Sanatoriums.

By GEORGE H. McMICHAEL, M.D., Buffalo, N.Y.

THERE are probably as many proposals for the solution of the liquor problem as there are sides to the question. As the evil is to some extent one of the peoples own choosing, it has been suggested that a popularly elected body for the control of the sale of intoxicants would have the effect of doing away with as much of it as would be found to be desirable. Some people profess to believe that the poor quality of the alcoholic beverages on sale in the saloons has much to do with what we know as excessive drinking, and they assert that by the prevention of adulteration and by the enforced maturing of spirits a great amount of inebriety might be stopped. This view is not held by scientific men and is not tenable, because it is the ethyl alcohol in all intoxicants which produces drunkenness, and well-matured whiskey is as powerful an intoxicant as any new liquor that can be obtained. Further, in some cases "bad" whiskey and "poor" beer are comparatively harmless as they have been largely diluted with water before being sold.

A second class of reformers says that no more need be done than simply to enforce the law as it at present exists, and drinking to excess would soon be a thing of the past. This remedy, however, involves a technical definition of what constitutes "excess," and it ignores the all-important fact that alcoholism is something more than a bad habit.

A certain number of persons advocate total prohibition of the sale of liquor whether the majority of the inhabitants wish it or not, and these gentlemen are not averse to a sacrifice of the opportunities of the many in order to save a few excessive drinkers. As is now well known, legislative experiments outside New York State have shown that prohibition has little practical result, and leads to a considerable amount of illicit drinking, which is obviously objectionable.

As the law now stands in this state (N.Y.), excessive drinkers are regarded as criminals, and are usually sent to prison, when they are not able to pay a fine. Some of them, however, are committed to the lunatic asylums now known as "State Hospitals," where they derive no more benefit than those sent to the penitentiary, because they receive neither medical nor moral treatment. They are simply detained for a period, and are then discharged as "not insane"; and they usually resume their drinking habits as soon as they are outside the asylum. Compulsory abstinence alone has never cured alcoholism—and never will. My idea of the detention of excessive drinkers,—reputable persons, who have not been guilty of any crime,—is not the idea carried out at the prison or the insane asylum. I propose to describe it in detail hereafter, but at this point I may mention the general mode of treatment which I have adopted for a number of years and which has proved eminently satisfactory. Briefly stated, it consists of suitable medical attention specially adapted to each case,—for there is no specific for alcoholism, any more than there is for other diseases of the nervous system,—proper diet, baths, and general hygienic measures, in addition to such moral influence as will control the patient's actions during his daily exercises, and semi-freedom until the time when he becomes able to take care of himself. The assistance of carefully selected attendants is, of course, necessary.

There are, and have been for years, a number of scientific men, chiefly physicians, who consider that the only way to deal with alcoholic inebriety is to face the undoubted truth that excessive drinking is a disease—not a mere bad habit—and that it can be cured by medical treatment, always provided that the patient is placed in a sanatorium where he will have proper care, suitable surroundings, and a satisfactory diet. For fully twenty years, the medical profession has told the public that there is a period in all excessive drinking when the vice becomes a disorder, when the alcoholic should be called a patient, and when he can no more be held capable of choice or of self-restraint in the matter of drinking than an epileptic can be supposed to be capable of avoiding a fit by an effort of will-power. Accordingly, it has been and is the recommendation of science and of medicine that the drinker should be cared for in a sanatorium where only this class of patients is treated. If I have repeated somewhat frequently the necessity of sanatorium treatment, I have done so in order to make it plain that alcoholism cannot be cured unless the patient lives in the house with the physician under whose care he is.

The law of the State of New York in regard to inebriety is most unsatisfactory. A statute is needed which would enable the friends of alcoholics to obtain medical treatment for them, if they are unwilling to submit voluntarily to it. The county judge or any supreme court judge, ought to be empowered to grant an order upon the certificate of any duly licensed physician, requiring the patient to remain in a sanatorium for one or two months, during which time he would be regarded as a sick man and treated as such. This method would stop the drinking to excess of a certain number of persons who cannot, at present, be restrained by law, and who persistently refuse medical aid in any form. They are unable to cease drinking of their own will, and yet they cannot believe that they are suffering from a serious disease. The "control" of which I have written should take the form of a suitable companion or nurse who would accompany the patient in his walks and daily exercises, so long as he was unable to take care of himself. My experience is that as soon as the excessive drinker realises his condition, and finds that his health has been benefited by his brief stay of a month or two at a sanatorium, he regains his normal self-control and feels and acts as a man among men. It is to the credit of alcoholics that when they are under treatment they pay every attention to the necessary details and are anxious to get well.

My belief is that a court order to compel an excessive drinker to take treatment would not be necessary in most cases, because I feel confident that just as soon as the state recognises that alcoholism is a disease, and provides a law for the advantage of those who suffer from it, the inebriate will understand the position in which he is placed, and will voluntarily accept the advice of his friends to take treatment at a sanatorium for his own good, and without resort to coercive measures. It is true that today many good men hesitate to place themselves in the hands of so-called "specialists" who profess to treat alcoholism, and the reason is not difficult to find. Experience has shown that these "specialists" give every patient the same stock prescription without individual attention or hygienic measures. In some instances the "treatment" consists simply of a certain number of hypodermic injections for a fixed number of days, in exchange for a certain amount of money. No words are strong enough to condemn this process, which has done so much to bring the treatment of alcoholism into disrepute. The persons who are guilty of the practice are not infrequently itinerants, and their stay in any town or city is usually short. It is not surprising that they have failed to secure the confidence of intelligent men. I would go so far as to suggest that sanatoriums for the treatment of alcoholism should be licensed by the state, and that such institutions should be regularly inspected,—perhaps four times a year. If this were done, good results would soon be obtained, and a permanent benefit would be quickly felt in every circle in which a patient had been cured.

Alcoholism is a disorder which kills its victim surely, but quite slowly, and during its course it brings much misery to the sufferer, to his family, and to the community in which he lives.

As it is certain that the children of excessive drinkers inherit a predisposition to fall victims to the disease to which their parents were predisposed before them, it is most important that these boys and girls should be protected from the example of a domestic environment in which drinking habits are prevalent. As we cannot at present interfere with the right of any man to marry and beget children, the proper remedy for the evil is to take the drinking parent away from his home until such time as he is cured, even if he is at first unwilling to be separated from his family.

I am probably well within the mark in saying that for one man who is born with the alcoholic susceptibility there are three who are educated to excessive drinking by their environment.

Fortunately, the educational influence of scientific treatment is almost certain not only to cure such men, but also to immensely increase their children's chances of overcoming the inherited predisposition.

If efforts were made to carry out successfully such a statute as I have suggested, time would undoubtedly prove the necessity of extending the period of detention of those for whom a few months' treatment are found insufficient. Still later, perhaps a statute might be necessary which would provide for the detention of all persons who have come before a court as a result of crimes committed under the influence of drink, or to which drunkenness has contributed, and for a similar detention of all offenders who have been convicted three times in any one year of acts of drunkenness which the law regards as offences. This system would be of permanent benefit to the patient himself and to the taxpayers, which is far more than can be said from the existing system. As I have already said, as the law now stands, the habitual drinker returns to his former way of living just as soon as he gets out of the penitentiary or the asylum.

The general effect upon the community of the scientific treatment of alcohol seen among all classes would be most beneficial. As a result, posterity would have to bear the greater part of the blame for its drinking habits, because the present generation would have taken the necessary precautions to cure, wherever possible, those who would be looked back upon as "drinking ancestors," if such method had been adopted.

New York State, the Empire State of the Union, ought not to lag behind in any matter which is of vital importance to the people. It is no exaggeration to say that there are no problems which equal the drink question in its far-reaching effects. The legislature ought to deal with the subject without delay, for the evil is increasing every day, and will continue to increase until some measures similar to the one outlined in this article is placed upon the statute book.

75 W. Tupper Street.

